# Structural insights into the production of 3-hydroxypropionic acid by aldehyde dehydrogenase from *Azospirillum brasilense*

**DOI:** 10.1038/srep46005

**Published:** 2017-04-10

**Authors:** Hyeoncheol Francis Son, Sunghoon Park, Tae Hyeon Yoo, Gyoo Yeol Jung, Kyung-Jin Kim

**Affiliations:** 1 School of Life Sciences, KNU Creative BioResearch Group, Kyungpook National University, Daehak-ro 80, Buk-ku, Daegu 702-701, Korea; 2 School of Energy and Chemical Engineering, Ulsan national Institute of Science and Technology (UNIST), Ulsan 44919, Korea; 3 Department of Molecular Science and Technology, Ajou University, Suwon 16499, Korea; 4 Department of Chemical Engineering and School of Interdisciplinary Bioscience and Bioengineering, Pohang University of Science and Technology, 77 Cheongam-ro, Nam-gu, Pohang, Gyeongbuk 37673, Korea

## Abstract

3-Hydroxypropionic acid (3-HP) is an important platform chemical to be converted to acrylic acid and acrylamide. Aldehyde dehydrogenase (ALDH), an enzyme that catalyzes the reaction of 3-hydroxypropionaldehyde (3-HPA) to 3-HP, determines 3-HP production rate during the conversion of glycerol to 3-HP. To elucidate molecular mechanism of 3-HP production, we determined the first crystal structure of a 3-HP producing ALDH, α-ketoglutarate-semialdehyde dehydrogenase from *Azospirillum basilensis (Ab*KGSADH), in its apo-form and in complex with NAD^+^. Although showing an overall structure similar to other ALDHs, the *Ab*KGSADH enzyme had an optimal substrate binding site for accepting 3-HPA as a substrate. Molecular docking simulation of 3-HPA into the *Ab*KGSADH structure revealed that the residues Asn159, Gln160 and Arg163 stabilize the aldehyde- and the hydroxyl-groups of 3-HPA through hydrogen bonds, and several hydrophobic residues, such as Phe156, Val286, Ile288, and Phe450, provide the optimal size and shape for 3-HPA binding. We also compared *Ab*KGSADH with other reported 3-HP producing ALDHs for the crucial amino acid residues for enzyme catalysis and substrate binding, which provides structural implications on how these enzymes utilize 3-HPA as a substrate.

3-Hydroxypropionic acid (3-HP) is one of top twelve value-added platform chemicals which can be produced from renewable biomass products^1^. 3-HP has diverse industrial applications in the production of such chemicals as acrylic acid, acrylamide, ethyl 3-HP, 3-hydroxymethyl-propionate, 3-hydroxypropionaldehyde, malonic acid, methylacrylate, 1,3-propanediol, propiolactone, and 3-HP- or acryl-based polymers^1^,^2^,^3^.

To date, two biosynthetic routes using glycerol or glucose as carbon substrate have been extensively studied for industrial production of 3-HP. With glucose as carbon source, 3-HP can be produced *via* malonyl-CoA or β-alanine^4^,^5^,^6^. With glycerol as substrate, glycerol is converted to 3-hydroxypropionaldehyde (3-HPA) by coenzyme B_12_-dependent glycerol dehydratase (DhaB) and 3-HPA is then converted to 3-HP by NAD^+^-dependent aldehyde dehydrogenases (ALDHs) (Fig. 1a)^7^,^8^. The route using glycerol as the carbon source is advantageous because the pathway is simple and cheap glycerol is abundantly available as a waste by product from biodiesel industry^9^,^10^,^11^,^12^,^13^.

Since 2008, eight 3-HP producing ALDHs have been characterized and these enzymes include DhaS from *Bacillus subtilis (Bs*DhaS)^14^, GapD4 from *Cupriavidus necator (Cn*GapD4)^15^, AldH from *Escherichia coli (Ec*AldH)^16^, PuuC from *Klebsiella pneumonia (Kp*PuuC)^17^, YdcW from *Klebsiella pneumonia (Kp*YdcW), YneI from *Klebsiella pneumonia (Kp*YneI)^18^, Ald4 from *Saccharomyces cerevisiae (Sc*Ald4)^19^, and α-ketoglutarate-semialdehyde dehydrogenase from *Azospirillum basilensis (Ab*KGSADH)^20^ (Fig. 1b). However none of them use 3-HPA as the physiological substrate. Thus, these ALDHs show low enzymatic activity, thus the conversion of 3-HPA to 3-HP is considered the rate-limiting step for the production of 3-HPA from glycerol. Furthermore, low ALDH activity has been reported to cause intracellular accumulation of highly toxic 3-HPA and this seriously hamper the cell growth and 3-HP production as well^21^,^22^,^23^,^24^,^25^. Although some studies to screen better ALDHs or to improve the existing ALDHs by protein engineering have been conducted, poor performance of ALDH still remains a significant challenge for successful 3-HP production^26^.

The ALDH family enzymes have been extensively studied and several crystal structures of ALDHs have also been determined. Amino acid sequence and structure analysis of various ALDHs revealed that this family of enzymes have different substrate specificities, although the overall structures of the enzymes are very similar. Among the eight ALDHs which have been tested for the conversion of 3-HPA to 3-HP, *Ab*KGSADH was first discovered as an enzyme that catalyzed the conversion of α-ketoglutarate-semialdehyde (α-KGSA) to α-ketoglutarate (α-KG) in an alternative pathway of _L_-arabinose metabolism^27^. In comparative studies, *Ab*KGSADH was identified as a highly efficient enzyme for the conversion of 3-HPA to 3-HP^20^. However, the crystal structures of 3-HP producing ALDH, including *Ab*KGSADH have not yet been reported, and the structural features that determine its 3-HPA binding ability have been veiled.

Here, we report the first crystal structure of the 3-HP producing ALDH from *A. basilensis (Ab*KGSADH) in its apo-form and in complex with NAD^+^ cofactor. On the basis of the docking simulation of 3-HPA binding to the *Ab*KGSADH active site and pertinent biochemical studies, we reveal the structural features of substrate specificity 3-HPA. We also analyzed the amino acids that constitute the substrate binding pockets of known 3-HP producing ALDHs. These studies may provide valuable structural information for the development of engineered ALDHs with high 3-HP producing activity.

## Results and Discussion

### Overall structure of *Ab*KGSADH

To elucidate the molecular mechanism of 3-HP producing ALDHs, we determined the crystal structure of *Ab*KGSADH at a 2.25 Å resolution. The refined structure was in good agreement with the X-ray crystallographic statistics for bond angles, bond lengths, and other geometric parameters (Table 1). The overall structure of *Ab*KGSADH shows a conventional conformation for the ALDH fold. The monomeric structure of *Ab*KGSADH consists of three domains: two core domains and one oligomerization domain (OGD) (Fig. 2a). The core domains consist of the N-terminal domain (NTD) (Met1-Arg123 and Val145-Leu253) and the C-terminal domain (CTD) (Gly254-Pro469). The NTD is composed of seven α-helices (α1–α7) and nine β-strands (β1–β4 and β7–β11), and forms the NAD(P)-binding Rossmann fold, where seven β-strands (β1–β2 and β7–β11) form a large β-sheet packed in the middle of the domain and other two β-strands (β3–β4) are located on the surface of the domain. The three α-helices (α1, α6 and α7) and the four α-helices (α2–α5) occupy both sides of the central β-sheet (Fig. 2a). The CTD consists of seven α-helices (α8–α14) and seven β-strands (β12–β18). Seven β-strands are also packed as a large β-sheet in the middle of the domain. Six α-helices surround the central β-sheet and one α-helix (α14) is located between the NTD and the OGD. The OGD (Val124-Pro144 and Tyr470-Val481) has two long β-strands (β6 and β19) and one short β-strand (β5), which are packed in a line and protrude from the NTD (Fig. 2a).

As observed in many other ALDH structures, *Ab*KGSADH forms a tetramer. Although there are two *Ab*KGSADH molecules in the asymmetric unit of our present structures, the tetrameric structure can be easily generated by one of the two folds from the P4_3_22 crystallographic symmetry operation (Fig. 2b). Dimerization is mainly mediated by the OGD and the CTD of *Ab*KGSADH. Three β-strands in the OGD and a β-sheet in the middle of the CTD form big β-sheet, and tetramerization of *Ab*KGSADH is mediated by the OGD and two α-helices (α2, α3) (Fig. 2b). Using the PISA software^28^, we calculated that a 5028.9 Å^2^ area of solvent accessible interface per monomer is buried, and the percentage of participating residues is 20.9%.

### NAD^+^ binding mode of *Ab*KGSADH

Previous research has indicated that the ALDH family of enzymes utilize NAD^+^ or NADP^+^ as a cofactor^29^. First, to identify the cofactor specificity of *Ab*KGSADH, we performed an ALDH activity assay using NAD^+^ and NADP^+^. *Ab*KGSADH showed less than 10% ALDH activity when NADP^+^ was used as a cofactor as compared to that when NAD^+^ was used (Fig. 3a). This result indicates that *Ab*KGSADH utilizes NAD^+^ as a cofactor instead of NADP^+^. To elucidate the cofactor binding mode of *Ab*KGSADH, we then determined the crystal structure of the protein in complex with NAD^**+**^ at a 2.6 Å resolution (Fig. 3b). The NAD^**+**^ cofactor is bound to an inter-domain space between the NTD and the CTD (Fig. 3c). The NAD^**+**^-binding pocket is constituted by seven loops (β7–α4, β8–α5, β10–α7, β11–β12, α8–β13, α9–α10, and α11–β16) and four α-helices (α4, α6, α7, and α10). The adenine ring is stabilized in the hydrophobic pocket that is formed by Phe151, Pro211, Ala212, Phe229, Val235 and Leu239, and a hydrogen bond with Ser215 also contributes to the binding of the ring. Residues Lys178, Glu181, and Pro211 constitute a suitable space for binding of the ribose ring, and stabilize the 2′-hydroxyl-group of the ring (Fig. 3d). The formation of the ribose ring binding site does not seem to be large enough to accommodate the phosphorylated ribose ring. This observation indicates that *Ab*KGSADH cannot utilize NADP^+^ as a cofactor, which is consistent with the results mentioned above. The pyrophosphate moiety is stabilized by residues Asn331, Arg333, and Arg334 through directly and water-mediated hydrogen bond networks. Residues Arg334 and Glu384 stabilize the ribose moiety of NAD^+^, and the nicotinamide ring is stabilized by residues Gln160 and Glu253 by hydrogen bonding (Fig. 3e).

### Substrate binding mode of *Ab*KGSADH

*Ab*KGSADH is known to utilize both α-ketoglutarate-semialdehyde (α-KGSA) and succinate-semialdehyde (SSA) as substrates^27^. To elucidate how *Ab*KGSADH accommodates these substrates, we performed molecular docking simulations of *Ab*KGSADH with α-KGSA and SSA. The molecular docking simulations revealed that these two substrates fit well into the somewhat positively charged substrate binding pocket (Fig. 4a). The aldehyde-groups of these substrates, which are the sites of enzyme reaction, are located in the same place around the catalytic residues (Fig. 4a). The aldehyde-group of α-KGSA is stabilized by Gln160 and Arg163 through hydrogen bonds, and two catalytic residues, Glu253 and Cys287, also assist the binding of the molecule (Fig. 4b). The 4′-keto-group of α-KGSA is stabilized by hydrogen bonds with Arg281, and the carboxyl-group of the molecule is stabilized by Glu106 and Gln160. The substrate binding pocket is also formed by several hydrophobic residues, such as Phe156, Val286, Ile288, Pro444, and Phe450, which seem to contribute to the stabilization of the hydrophobic part of α-KGSA (Fig. 4b). The binding of SSA is similar to that of α-KGSA, however, the stabilization of the carboxyl-group of SSA is quite different. Arg281, a residue that is involved in the stabilization of the 4′-keto-group of α-KGSA, forms a hydrogen bond with the carboxyl-group of SSA instead (Fig. 4c). These observations explain how *Ab*KGSADH can accommodate both α-KGSA and SSA as real substrates.

### 3-HPA binding mode of *Ab*KGSADH

To elucidate the 3-HPA binding mode of *Ab*KGSADH, we attempted to determine the crystal structure of *Ab*KGSADH in complex with 3-HPA substrate or the 3-HP product, however, both cocrystallization and soaking experiments were unsuccessful. The molecular docking simulation of *Ab*KGSADH with 3-HPA did allow us to speculate how *Ab*KGSADH accommodates an unnatural substrate 3-HPA. The 3-HPA molecule is bound at the same position as the α-KGSA and SSA molecules (Fig. 4a). Moreover, the aldehyde-group of 3-HPA is located in the same position as those of α-KGSA and SSA, and is stabilized by the same residues (Fig. 4a). The 3′-hydroxyl-group of 3-HPA is stabilized by residues Asn159, Gln160, and Arg163 through hydrogen bonds, and of these three residues, Gln160 and Arg163 are also involved in the binding of the aldehyde-group of 3-HPA (Fig. 4d). As observed in the binding of α-KGSA and SSA, hydrophobic residues, such as Phe156, Val286, Ile288, and Phe450, seem to also contribute to stabilization of the hydrophobic part of 3-HPA (Fig. 4d). One interesting observation is that Arg281, which is a crucial residue for the binding of α-KGSA and SSA, is located distal from the bound 3-HPA and does not participate in its stabilization (Fig. 4d). We speculate that this observation is derived from the fact that 3-HPA has two and one fewer carbon than α-KGSA and SSA, respectively.

To confirm the involvement of these residues in the enzyme catalysis and the binding of the 3-HPA substrate, we then performed site-directed mutagenesis experiments. First, we mutated the two catalytic residues, Glu253 and Cys287, to alanine residues, and observed that these mutants exhibited an almost complete loss of enzyme activity (Fig. 4e), indicating that *Ab*KGSADH has the same enzymatic mechanism as other ALDH family enzymes. Second, we mutated residues that form hydrogen bonds with the aldehyde- and hydroxyl-group of 3-HPA to alanine residues. The *Ab*KGSADH^R163A^ mutant showed 90% of the enzyme activity that was present with the wild-type enzyme (Fig. 4e), and the result indicates that 3-HPA can be stabilized sufficiently by hydrogen bonding with other residues even without Arg163. Interestingly, the *Ab*KGSADH^N159A^ and the *Ab*KGSADH^Q160A^ mutants showed approximately 30% higher enzyme activities than the wild-type enzyme (Fig. 4e). These results imply that there is no significant issue in stabilizing 3-HPA even if one of the hydrogen bonding residues is absent, as observed in the *Ab*KGSADH^R163A^ mutant. Rather, substitutions of Asn159 and Gln160 to alanine seem to increase the hydrophobicity of the substrate binding site and consequently increased the stabilization of 3-HPA. Finally, we mutated residues involved in the constitution of the hydrophobic substrate binding site to alanine residues. All of these mutants, *Ab*KGSADH^F156A^, *Ab*KGSADH^V286A^, *Ab*KGSADH^I288A^, and *Ab*KGSADH^F450A^, exhibited decreased or complete loss of activities compared with the wild-type enzyme (Fig. 4e). We propose that the formation of substrate binding sites with optimal size and shape by these hydrophobic residues is very important for accommodating 3-HPA as a substrate.

### Comparison of 3-HP producing ALDHs

**Figure 1:**
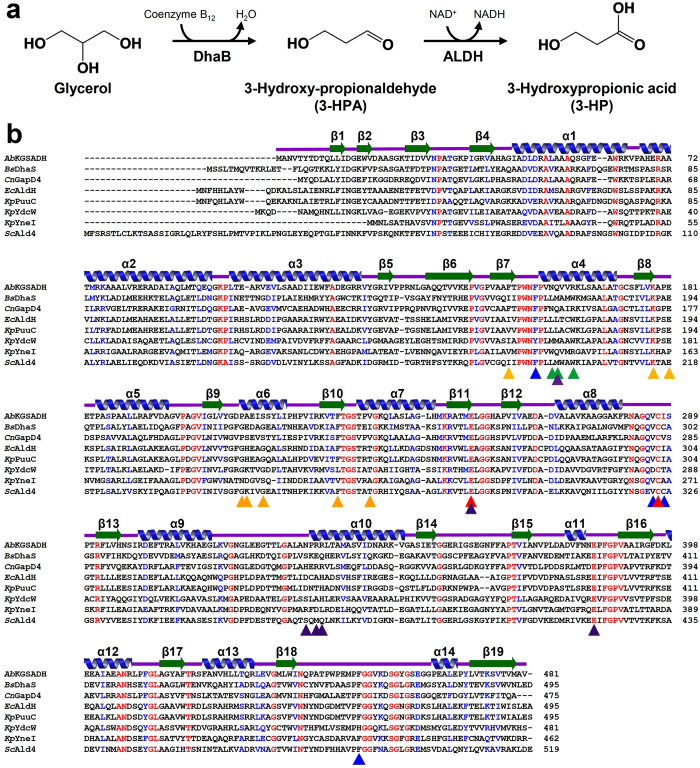
3-HP production pathway and amino acid sequence alignment of 3-HP producing ALDHs. (**a**) 3-HP production pathway. (**b**) Amino acid sequence alignment of eight reported 3-HP producing ALDHs. The secondary structure elements are drawn based on the structure of *Ab*KGSADH. The catalytic residues of *Ab*KGSADH are indicated by red colored triangles, and the residues involved in the binding of aldehyde- and hydroxyl-groups of 3-HPA are indicated by green colored triangles. The residues involved in the formation of hydrophobic pocket are indicated by blue colored triangles. The residues involved in the adenine ring and 2′-hydroxyl-group of NAD^+^ are indicated by orange colored residues, and the residues involved in the formation of pyrophosphate, ribose ring, and nicotinamide are indicated by purple colored triangles. *Ab, Bs, Cn, Ec, Kp,* and *Sc* are abbreviations of *Azospirillum basilensis, Bacillus subtilis, Cupriavidus necator, Escherichia coli, Klebsiella pneumonia,* and *Saccharomyces cerevisiae*, respectively.

**Figure 2:**
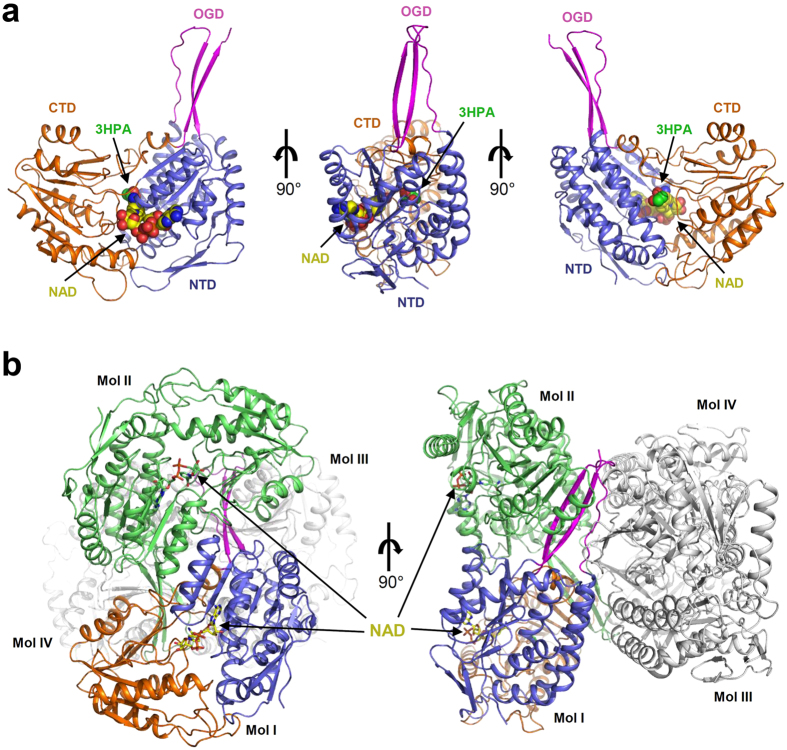
Overall structure of *Ab*KGSADH. (**a**) The monomeric structure of *Ab*KGSADH. The monomeric structure of *Ab*KGSADH is presented as a cartoon diagram. N-terminal domain (NTD), C-terminal domain (CTD), and oligomerization domain (OGD) are distinguished with light-blue, orange, and magenta colors, respectively, and labeled. The bound NAD^+^ from the crystal data and 3-HPA derived from molecular docking simulation are shown as sphere models with yellow and green colors, respectively. The left and right figures are rotated 90 degree vertically from the figure in the middle. (**b**) Tetrameric structure of *Ab*KGSADH. The tetrameric structure of *Ab*KGSADH is presented as a cartoon diagram. Mol I is presented with colors of light-blue, orange, and magenta for NTD, CTD, and OGD, respectively, and Mol II is presented with green color. The other two molecules are shown with grey colors. The bound NAD^+^ is presented as stick models with yellow color. The right figure is rotated by 90 degree vertically from the left figure.

**Figure 3:**
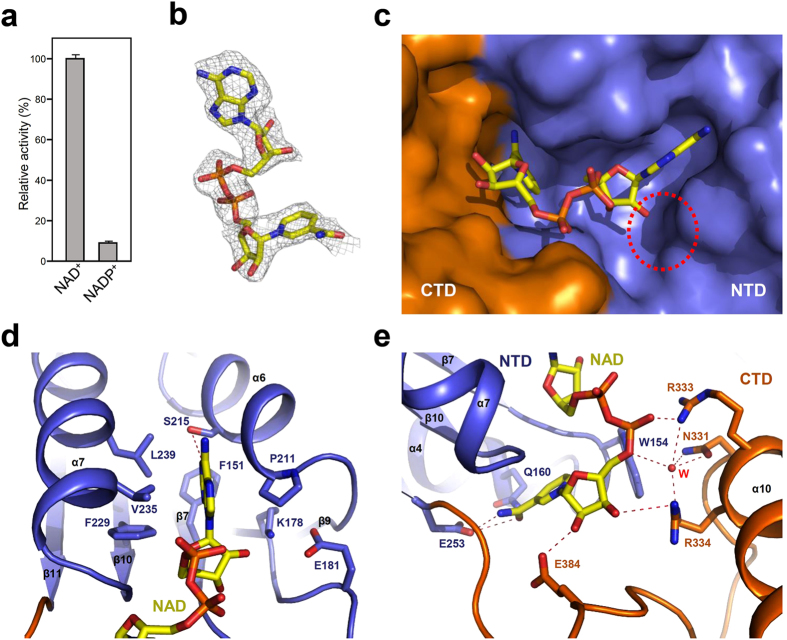
NAD^+^ cofactor binding mode of *Ab*KGSDH. (**a**) Relative activity of NAD^+^ and NADP^+^ in *Ab*KGSADH (**b**) Electron density map of the bound NAD^+^ in *Ab*KGSADH. The Fo-Fc electron density map of bound NAD^+^ is shown with a gray-colored mesh, and contour 2.5 σ. The NAD^+^ is shown as stick model with yellow color. (**c**) NAD^+^ binding pocket of *Ab*KGSADH. The *Ab*KGSADH structure is shown as a surface model. The NTD and the CTD are distinguished with light-blue and orange colors, respectively. The bound NAD^+^ is shown as a stick model with yellow color. The red dotted circle indicates the binding pocket of the 2′-hydroxyl-group of *Ab*KGSADH. (**d**,**e**) NAD^+^ cofactor binding mode of *Ab*KGSDH. The binding mode of adenine ring and 2′-hydroxyl-group (**d**), and pyrophosphate, ribose ring, and nicotinamide ring (**e**) of *Ab*KGSADH. The structure of *Ab*KGSADH is shown as a cartoon diagram. The NTD and the CTD are distinguished with light-blue and orange colors, respectively. The residues involved in the NAD^+^ binding are shown as stick models and labeled appropriately. One water molecule involved in the stabilization of NAD^+^ is shown as a sphere model with a red color. The bound NAD^+^ is shown as a stick model with a yellow color. Red color dotted lines indicate hydrogen bonds contributing to of NAD^+^ binding.

**Figure 4:**
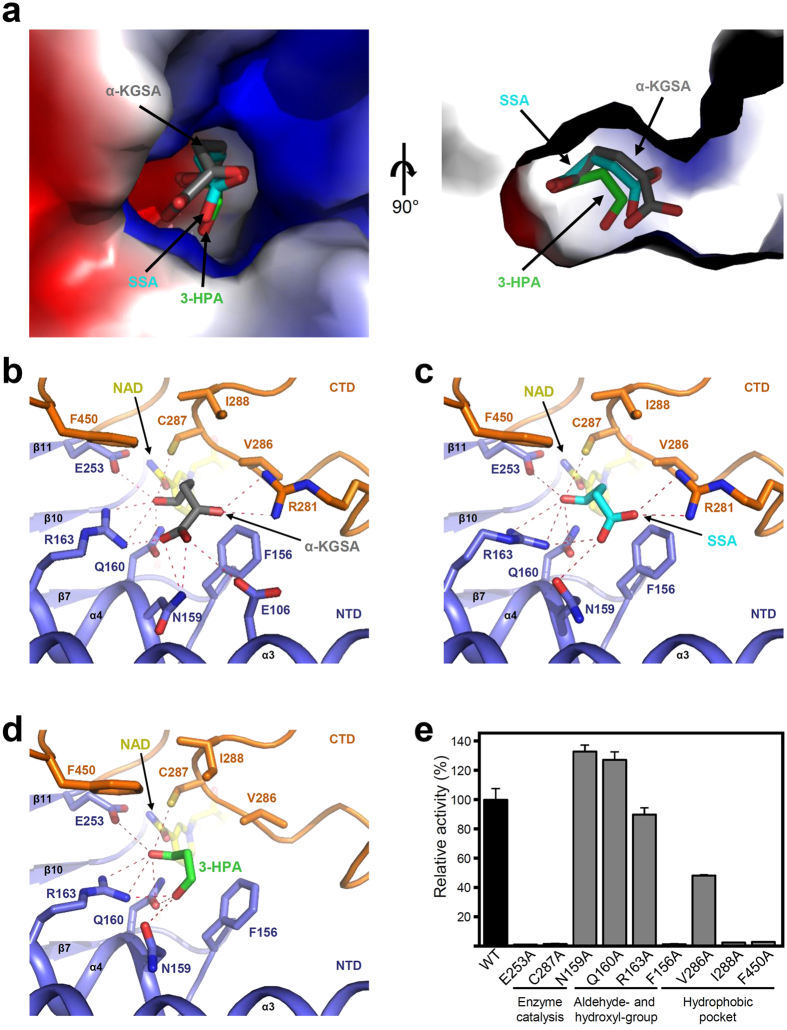
Substrate binding mode of *Ab*KGSADH. (**a**) Electrostatic potential surface presentation of substrate binding mode of *Ab*KGSADH. The *Ab*KGSADH structure is shown as an electrostatic potential surface presentation. The binding mode of α-ketoglutarace semialdehyde (α-KGSA), succinate semialdehyde (SSA), and 3-hydroxypropionaldehyde (3-HPA) is predicted by molecular docking simulation. α-KGSA, SSA, and 3-HPA are presented by stick models with grey, cyan, and green colors, respectively. The right figure is rotated 90 degrees vertically from the left figure. (**b**,**c**,**d**) Substrate binding mode of *Ab*KGSADH. The substrate binding mode of α-KGSA (**b**), SSA (**c**), and 3-HPA (**d**) in *Ab*KGSADH. The *Ab*KGSADH structure is shown as a cartoon diagram. The NTD, and the CTD is distinguished with light-blue and orange colors, respectively. The residues involved in the substrate binding are shown as stick models and labeled appropriately. Secondary structure elements are labeled. α-KGSA, SSA, and 3-HPA are shown as stick models with grey, cyan, and green colors, respectively. Hydrogen bonds involved in the substrate binding are shown with red-colored dotted lines. (**e**) Site-directed mutagenesis of *Ab*KGSADH. Residues involved in binding of the 3-HPA substrate are replaced by alanine residues. The relative activities of recombinant mutant proteins were measured and compared with that of wild-type *Ab*KGSADH.

So far, eight 3-HP producing ALDHs, including *Ab*KGSADH, have been reported. To structurally analyse how these enzymes utilize 3-HPA as a substrate, we compared the key residues of *Ab*KGSADH in enzyme catalysis and substrate binding for 3-HPA with those of the other seven 3-HP producing ALDHs (Fig. 1b, Table 2). The amino acid sequence similarities within eight 3-HP producing ALDHs are 31% to 83%, and those between *Ab*KGSADH and the other seven 3-HP producing ALDHs are 32% to 50%. As expected, the two catalytic residues, Glu253 and Cys287, in *Ab*KGSADH are completely conserved in all 3-HP producing ALDHs. However, the three residues involved in the stabilization of the aldehyde- and hydroxyl-group of 3-HPA, Asn159, Gln160, and Arg163 in *Ab*KGSADH, are variable in other 3-HP producing ALDHs (Fig. 1b, Table 2). Combined with the previously described results that mutations of these residues to alanine did not significantly affect enzyme activity, we propose that, for the stabilization of the aldehyde- and hydroxyl-groups of 3-HPA, the combination of several residues rather than a particular residue is important. Interestingly, the four hydrophobic residues forming the hydrophobic pockets, Phe156, Val286, Ile288, and Phe450 in *Ab*KGSADH, are highly conserved throughout all 3-HP producing ALDHs (Fig. 1b, Table 2). As described above, a single amino acid mutation of any of these four hydrophobic residues to alanine showed decreased or almost complete loss of enzyme activity (Fig. 4e). Taken together, we propose that formation of hydrophobic pockets with optimal size and shape is critical for 3-HP producing ALDHs to accept 3-HPA as a substrate.

In summary, we report the first crystal structure of the 3-HP producing ALDH, *Ab*KGSADH, and provide structural insight into how the 3-HP producing ALDHs utilize the unnatural substrate 3-HPA. For accepting 3-HPA, the location of the appropriate residues for hydrogen bonding to the aldehyde- and the hydroxyl-groups of 3-HPA is important. Moreover, a hydrophobic pocket with optimal size and shape to bind the hydrophobic portion of 3-HPA is also critical. This structural information might be used for developing 3-HP producing ALDHs that possess a higher 3-HP production activity.

## Methods

### Cloning, expression and purification of *Ab*KGSADH

The *Ab*KGSADH coding gene was amplified through polymerase chain reaction (PCR) using synthetic gene in a pBHA vector by Bioneer. The PCR products were digested by NdeI and XhoI restriction enzymes, and sub-cloned into the pProEX-HTa expression vector (Thermo Fisher Scientific) which contained a 6xHis tag and rTEV protease cleavage site at the N-terminus of the target protein. The pProEX-HTa:*Ab*KGSADH was transformed into a *E. coli* BL21(DE3)-T1^R^ strain, which was grown to an OD_600_ of 0.6 in LB medium containing 100 mg L^−1^ ampicillin at 310 K and *Ab*KGSADH protein expression was induced by 0.5 mM 1-thio-β-D-galatopyranoside (IPTG). After 20 h at 293 K, the cell were harvested by centrifugation at 4,000× g for 15 min at 277 K. The cell pellet was resuspended in ice-cold buffer A (40 mM Tris-HCl pH 8.0) and disrupted by ultrasonication. The cell debris was removed by centrifugation at 13,000 g for 30 min, and the lysate was applied onto a Ni-NTA agarose column (Qiagen). After washing with buffer B (40 mM Tris-HCl pH 8.0 and 25 mM Imidazole), the bound proteins were eluted with buffer C (40 mM Tris-HCl pH 8.0 and 300 mM Imidazole). Finally, trace amounts of contaminants were removed by size-exclusive chromatography using Sephacryl S-300 prep-grade column (320 ml, GE Healthcare) equilibrated with buffer A. The eluted protein had a molecular weight of about 200 kDa, indicating a tetrameric structure. The protein was concentrated to 50 mg mL^−1^ using a spin column (Amicon Ultra Centrifugal Filter, 30 kDa pore size), and kept at 193 K for further experiments. All purification steps were performed at 277 K.

### Crystallization and data collection of *Ab*KGSADH

Crystallization of the purified *Ab*KGSADH protein was initially tried with commercially available sparse-matrix screens, including Index, PEG ion I and II (Hampton Research), and Wizard Classic I and II (Rigaku Reagents), using the sitting-drop vapor diffusion method on the MRC Crystallization plates (Molecular Dimensions) at 295 K. Each experiment consisted of mixing 1.0 μL protein solution (60 mg mL^−1^, 40 mM Tris-HCl pH 8.0) with 1.0 μL reservoir solution and then equilibrating against 50 μL reservoir solution. *Ab*KGSADH crystals were observed from several crystallization screening conditons. After several steps of crystal improvement, the best quality crystals appeared in 16% polyethylene glycol 3350, 0.1 M sodium cacodylate pH 6.5, and 0.2 M Magnesium chloride hexahydrate. The crystals were transferred to cryoprotectant solution containing 25% polyethylene glycol 3350, 0.1 M sodium cacodylate pH 6.5, 0.2 M Magnesium chloride hexahydrate, and 30% (v/v) glycerol. The crystals were fished out with a loop larger than the crystals and flash-frozen by immersion in liquid nitrogen at 100 K. Data were collected to a maximum resolution on the detector of 2.18 Å at 7A beamline of the Pohang Acclerator Laboratory (PAL, Pohang, Korea), using a Quantum 270 CCD detector (ADSC, USA). All data were indexed, integrated, and scaled together using the HKL2000 software package^30^. The crystals of *Ab*KGSADH belonged to the space group P4_3_22 with unit cell parameters a = b = 129.12 Å, c = 118.12 Å, α = β = γ = 90° Assuming two *Ab*KGSADH molecules in asymmetric unit, the crystal volume per unit of protein mass was 2.46 Å^3^ Da^−1^, which means the solvent content was approximately 50%^31^.

### Structure determination of *Ab*KGSADH

The structure of apo-form of *Ab*KGSADH was determined by molecular replacement with the CCP4 version of MOLREP^32^, using the structure of Succinic-semialdehyde dehydrogenase (SSADH) from *Homo sapiens* (PDB code 2W8R) as a search model. Further model building was performed manually using the program WinCoot^33^, and refinement was performed with CCP4 refmac5^34^. The structure of *Ab*KGSADH in complex with NAD^+^ was solved by molecular replacement using the crystal structure of the apo-form of *Ab*KGSADH. The data statistics are summarized in Table 1. The refined model of the apo-form of *Ab*KGSADH and that in complex with NAD^+^ were deposited in the Protein Data Bank with PDB codes of 5X5T and 5X5U, respectively.

### Molecular docking simulations of *Ab*KGSADH

Molecular docking simulations of α-KGSA, SSA and 3-HPA to *Ab*KGSADH structure were performed by AutoDock Vina software^35^. *Ab*KGSADH structure in complex with NAD^+^ cofactor (PDB code of 5X5U) and the α-KGSA, SSA and 3-HPA ligands were prepared using the JLigand software. For the docking simulation, the *pdbqt* files were generated using AutoDock Tools, and all steps for simulation and grid box creation were performed according to the AutoDock Vina manual. The grid size for α-KGSA was x = 40, y = 50, z = 36, and grid center was designated at x = −35.377, y = −49.707, z = −4.816. And the grid size for SSA was x = 28, y = 50, z = 34, and grid center was designated at x = −37.77, y = −49.853, z = −4.29. Last, the grid size for 3-HPA was x = 26, y = 32, z = 34, and grid center was designated at x = −37.903, y = −46.826, z = −4.534. The final conformations produced in this simulation were checked using PyMOL software.

### Activity assay of *Ab*KGSADH

The activity of *Ab*KGSADH was determined by measuring the increase of absorbance at 340 nm (extinction coefficient of 6.22 × 10^3^ M^−1^ cm^−1^). Enzyme reaction was performed with a reaction mixture of 1 mL total volume at 303 K. The reaction mixture contained 100 mM Tris-HCl, pH 8.0, 10 mM 3-HPA, and 1 mM NAD(P), and the background rate of the assay in the absence of enzyme is zero. The reaction was initiated by the addition of enzyme to a final concentration of 200 nM. The *Ab*KGSADH activity assay was performed in duplicate reaction.

## Additional Information

**How to cite this article**: Son, H. F. *et al*. Structural insights into the production of 3-hydroxypropionic acid by aldehyde dehydrogenase from *Azospirillum brasilense. Sci. Rep.* **7**, 46005; doi: 10.1038/srep46005 (2017).

**Publisher's note:** Springer Nature remains neutral with regard to jurisdictional claims in published maps and institutional affiliations.

## Figures and Tables

**Table 1:** Data collection and refinement statistics.

	*Ab*KGSADH_Apo	*Ab*KGSADH_NAD^+^
Data collection		
* *Space group	P4_3_22	P4_3_22
Cell dimensions		
* a, b, c* (Å)	129.04, 129.04, 118.09	129.3, 129.3, 118.36
* *α, β, γ (°)	90.00, 90.00, 90.00	90.00, 90.00, 90.00
* *Resolution (Å)	50.00–2.25 (2.29–2.25)	50.00–2.30 (2.34–2.30)
* R*_sym_ or *R*_merge_	12.1 (29.8)	9.2 (26.2)
* I*/σ(*I*)	27.63 (5.23)	66.47 (21.03)
* *Completeness (%)	95.1 (95.1)	95.2 (100.0)
* *Redundancy	3.4 (3.2)	13.7 (13.1)
Refinement		
* *Resolution (Å)	50.00–2.25	50.00–2.30
* *No. reflections	43134	40594
* R*_work_/*R*_free_	17.4 (24.0)	16.2 (22.7)
* *No. atoms	7528	7748
* *Protein	7096	7096
* *Ligand/ion	6	94
* *Water	426	558
* B*-factors	30.362	22.21
* *Protein	30.32	21.86
* *Ligand/ion	38.60	40.00
* *Water	33.43	27.78
R.m.s. deviations		
* *Bond lengths (Å)	0.0163	0.0171
* *Bond angles (°)	1.7737	1.8604

^*^Values in parentheses are for highest-resolution shell.

**Table 2:** Comparison of key amino acids involved in the enzyme catalysis and the substrate binding sites among the 3-HP producing ALDHs.

Residue number in *Ab*KGSADH	Enzyme catalysis		Aldehyde- and hydroxyl-group			Hydrophobic pocket			
	253	287	159	160	163	156	286	288	450
*Ab*KGSADH	E	C	N	Q	R	F	V	I	F
*Bs*DhaS	E	C	L	M	W	F	V	C	F
*Cn*GapD4	E	C	N	Q	R	F	V	V	F
*Ec*AldH	E	C	L	L	W	F	V	I	F
*Kp*PuuC	E	C	L	L	W	F	V	I	F
*Kp*YdcW	E	C	L	M	W	Y	D	T	H
*Kp*YneI	E	C	V	Q	R	F	V	A	F
*Sc*Ald4	E	C	L	M	W	F	V	C	F

## References

[b1] Werpy T. & P. G. Top value added chemicals from biomass (2004).

[b2] Kumar V., Ashok S. & Park S. Recent advances in biological production of 3-hydroxypropionic acid. Biotechnol Adv 31, 945–961, doi: 10.1016/j.biotechadv.2013.02.008 (2013). 23473969

[b3] Valdehuesa K. N. G. . Recent advances in the metabolic engineering of microorganisms for the production of 3-hydroxypropionic acid as C3 platform chemical. Appl Microbiol Biot 97, 3309–3321, doi: 10.1007/s00253-013-4802-4 (2013). 23494623

[b4] Hugler M., Menendez C., Schagger H. & Fuchs G. Malonyl-coenzyme A reductase from Chloroflexus aurantiacus, a key enzyme of the 3-hydroxypropionate cycle for autotrophic CO2 fixation. Journal of bacteriology 184, 2404−+, doi: 10.1128/Jb.184.9.2404-2410.2002 (2002). 11948153 PMC134993

[b5] Hugler M., Huber H., Stetter K. O. & Fuchs G. Autotrophic CO2 fixation pathways in archaea (Crenarchaeota). Arch Microbiol 179, 160–173, doi: 10.1007/s00203-002-0512-5 (2003). 12610721

[b6] Rathnasingh C. . Production of 3-hydroxypropionic acid via malonyl-CoA pathway using recombinant Escherichia coli strains. J Biotechnol 157, 633–640, doi: 10.1016/j.jbiotec.2011.06.008 (2012). 21723339

[b7] Ashok S., Raj S. M., Rathnasingh C. & Park S. Development of recombinant Klebsiella pneumoniae Delta dhaT strain for the co-production of 3-hydroxypropionic acid and 1,3-propanediol from glycerol. Appl Microbiol Biot 90, 1253–1265, doi: 10.1007/s00253-011-3148-z (2011). 21336929

[b8] Forage R. G. & Foster M. A. Glycerol fermentation in Klebsiella pneumoniae: functions of the coenzyme B12-dependent glycerol and diol dehydratases. Journal of bacteriology 149, 413–419 (1982). 7035429 10.1128/jb.149.2.413-419.1982 PMC216523

[b9] Raj S. M., Rathnasingh C., Jung W. C. & Park S. Effect of process parameters on 3-hydroxypropionic acid production from glycerol using a recombinant Escherichia coli. Appl Microbiol Biot 84, 649–657, doi: 10.1007/s00253-009-1986-8 (2009). 19352643

[b10] Toraya T. . Mechanism-based inactivation of coenzyme B12-dependent diol dehydratase by 3-unsaturated 1,2-diols and thioglycerol. Journal of biochemistry 144, 437–446, doi: 10.1093/jb/mvn086 (2008). 18586770

[b11] Forrest A. K., Sierra R. & Holtzapple M. T. Effect of biodiesel glycerol type and fermentor configuration on mixed-acid fermentations. Bioresource Technol 101, 9185–9189, doi: 10.1016/j.biortech.2010.07.041 (2010). 20674340

[b12] Nitayavardhana S. & Khanal S. K. Biodiesel-derived crude glycerol bioconversion to animal feed: A sustainable option for a biodiesel refinery. Bioresource Technol 102, 5808–5814, doi: 10.1016/j.biortech.2011.02.058 (2011). 21382713

[b13] da Silva G. P., Mack M. & Contiero J. Glycerol: A promising and abundant carbon source for industrial microbiology. Biotechnol Adv 27, 30–39, doi: 10.1016/j.biotechadv.2008.07.006 (2009). 18775486

[b14] Su M., Li Y., Ge X. & Tian P. 3-Hydroxypropionaldehyde-specific aldehyde dehydrogenase from Bacillus subtilis catalyzes 3-hydroxypropionic acid production in Klebsiella pneumoniae. Biotechnology letters 37, 717–724, doi: 10.1007/s10529-014-1730-z (2015). 25409630

[b15] Chu H. S. . Metabolic engineering of 3-hydroxypropionic acid biosynthesis in Escherichia coli. Biotechnology and bioengineering 112, 356–364, doi: 10.1002/bit.25444 (2015). 25163985

[b16] Jo J. E. . Cloning, expression, and characterization of an aldehyde dehydrogenase from Escherichia coli K-12 that utilizes 3-Hydroxypropionaldehyde as a substrate. Appl Microbiol Biot 81, 51–60, doi: 10.1007/s00253-008-1608-x (2008). 18668238

[b17] Raj S. M., Rathnasingh C., Jung W. C., Selvakumar E. & Park S. A Novel NAD(+)-dependent Aldehyde Dehydrogenase Encoded by the puuC Gene of Klebsiella pneumoniae DSM 2026 that Utilizes 3-Hydroxypropionaldehyde as a Substrate. Biotechnol Bioproc E 15, 131–138, doi: 10.1007/s12257-010-0030-2 (2010).

[b18] Luo L. H. . Identification and characterization of Klebsiella pneumoniae aldehyde dehydrogenases increasing production of 3-hydroxypropionic acid from glycerol. Bioprocess and biosystems engineering 36, 1319–1326, doi: 10.1007/s00449-012-0880-4 (2013). 23297067

[b19] Li Y., Su M., Ge X. & Tian P. Enhanced aldehyde dehydrogenase activity by regenerating NAD+ in Klebsiella pneumoniae and implications for the glycerol dissimilation pathways. Biotechnology letters 35, 1609–1615, doi: 10.1007/s10529-013-1243-1 (2013). 23794046

[b20] Ko Y., Ashok S., Zhou S., Kumar V. & Park S. Aldehyde dehydrogenase activity is important to the production of 3-hydroxypropionic acid from glycerol by recombinant Klebsiella pneumoniae. Process Biochem 47, 1135–1143, doi: 10.1016/j.procbio.2012.04.007 (2012).

[b21] Lim H. G., Noh M. H., Jeong J. H., Park S. & Jung G. Y. Optimum Rebalancing of the 3-Hydroxypropionic Acid Production Pathway from Glycerol in Escherichia coli. ACS synthetic biology 5, 1247–1255, doi: 10.1021/acssynbio.5b00303 (2016). 27056171

[b22] Barbirato F., Soucaille P. & Bories A. Physiologic Mechanisms Involved in Accumulation of 3-Hydroxypropionaldehyde during Fermentation of Glycerol by Enterobacter agglomerans. Applied and environmental microbiology 62, 4405–4409 (1996). 16535461 10.1128/aem.62.12.4405-4409.1996 PMC1388999

[b23] Celinska E. Debottlenecking the 1,3-propanediol pathway by metabolic engineering. Biotechnol Adv 28, 519–530, doi: 10.1016/j.biotechadv.2010.03.003 (2010). 20362657

[b24] Barbirato F., Grivet J. P., Soucaille P. & Bories A. 3-Hydroxypropionaldehyde, an inhibitory metabolite of glycerol fermentation to 1,3-propanediol by enterobacterial species. Applied and environmental microbiology 62, 1448–1451 (1996). 8919810 10.1128/aem.62.4.1448-1451.1996 PMC167915

[b25] Kumar V., Ashok S. & Park S. Recent advances in biological production of 3-hydroxypropionic acid. Biotechnol Adv 31, 945–961, doi: 10.1016/j.biotechadv.2013.02.008 (2013). 23473969

[b26] Saxena R. K., Anand P., Saran S. & Isar J. Microbial production of 1,3-propanediol: Recent developments and emerging opportunities. Biotechnol Adv 27, 895–913, doi: 10.1016/j.biotechadv.2009.07.003 (2009). 19664701

[b27] Watanabe S., Kodaki T. & Makino K. A novel alpha-ketoglutaric semialdehyde dehydrogenase Evolutionary insight into an alternative pathway of bacterial L-arabinose metabolism. Journal of Biological Chemistry 281, 28876–28888, doi: 10.1074/jbc.M602585200 (2006). 16835232

[b28] Krissinel E. & Henrick K. Inference of macromolecular assemblies from crystalline state. Journal of molecular biology 372, 774–797, doi: 10.1016/j.jmb.2007.05.022 (2007). 17681537

[b29] Sophos N. A. & Vasiliou V. Aldehyde dehydrogenase gene superfamily: the 2002 update. Chem-Biol Interact 143, 5–22, doi: Pii S0009-2797(02)00163-1 doi: 10.1016/S0009-2797(02)00163-1 (2003). 12604184

[b30] Otwinowski Z. & Minor W. Processing of X-ray diffraction data collected in oscillation mode. Method Enzymol 276, 307–326, doi: 10.1016/S0076-6879(97)76066-X (1997). 27754618

[b31] Matthews B. W. Solvent content of protein crystals. Journal of molecular biology 33, 491–497 (1968). 5700707 10.1016/0022-2836(68)90205-2

[b32] Vagin A. & Teplyakov A. Molecular replacement with MOLREP. Acta crystallographica. Section D, Biological crystallography 66, 22–25, doi: 10.1107/S0907444909042589 (2010). 20057045

[b33] Emsley P. & Cowtan K. Coot: model-building tools for molecular graphics. Acta crystallographica. Section D, Biological crystallography 60, 2126–2132, doi: 10.1107/S0907444904019158 (2004). 15572765

[b34] Murshudov G. N., Vagin A. A. & Dodson E. J. Refinement of macromolecular structures by the maximum-likelihood method. Acta crystallographica. Section D, Biological crystallography 53, 240–255, doi: 10.1107/S0907444996012255 (1997). 15299926

[b35] Trott O. & Olson A. J. AutoDock Vina: improving the speed and accuracy of docking with a new scoring function, efficient optimization, and multithreading. J Comput Chem 31, 455–461, doi: 10.1002/jcc.21334 (2010). 19499576 PMC3041641

